# Supplementary material for: Network subgraph-based approach for analyzing and comparing molecular networks
Source: PeerJ. 2022 May 3;10:e13137. doi: 10.7717/peerj.13137 (PMC9074881; doi:10.7717/peerj.13137)
Supplement: File S6 [file peerj-10-13137-s006.docx]

To address the concern why our method can distinguish networks with similar global topology, we provide a detail discussion in below. We plot the cumulative probability functions (‘cpfs’) of the 3-node subgraphs for the first trial; so there are 18 ‘cpfs’. As shown in the Figure S6.1, the small-world and scale-free networks ‘cpfs’ can be clearly distinguished from the rest. Among the other four random networks, the ‘cpfs’ of aging random network and citation random network are quite close but with minor difference, as shown in Figure S6.2; thus, indicates the effectiveness of our method.


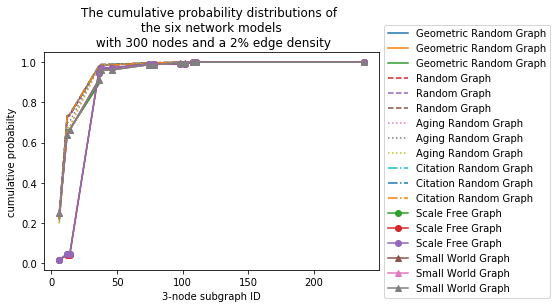


Figure S6.1. The small-world and scale-free networks ‘cpfs’ can be clearly distinguished from the rest.


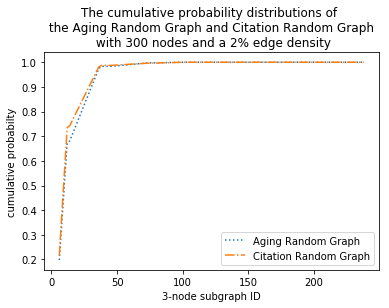


Figure S6.2. The ‘cpfs’ of aging random network and citation random network are quite close but with minor difference.
