# Supplementary figures and images for: Network subgraph-based approach for analyzing and comparing molecular networks
Source: PeerJ. 2022 May 3;10:e13137. doi: 10.7717/peerj.13137 (PMC9074881; doi:10.7717/peerj.13137)

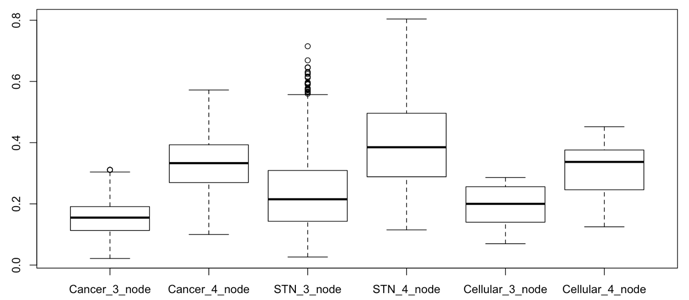

Supplement: File S8 [file peerj-10-13137-s008.png]
